# Recent advances in the clinical spectrum and pathomechanisms associated with X-linked myopathy with excessive autophagy and other *VMA21*-related disorders

**DOI:** 10.1177/22143602251314767

**Published:** 2025-03-04

**Authors:** Ilaria Cocchiararo, Perrine Castets

**Affiliations:** 1 Department of Cell Physiology and Metabolism, Faculty of Medicine, University of Geneva, Geneva, Switzerland

**Keywords:** autophagic vacuolar myopathies, VMA21, X-linked myopathy with excessive autophagy

## Abstract

X-linked myopathy with excessive autophagy (XMEA) is a rare neuromuscular disorder caused by mutations in the *VMA21* gene, encoding a chaperone protein present in the endoplasmic reticulum (ER). In yeast and human, VMA21 has been shown to chaperone the assembly of the vacuolar (v)-ATPase proton pump required for the acidification of lysosomes and other organelles. In line with this, VMA21 deficiency in XMEA impairs autophagic degradation steps, which would be key in XMEA pathogenesis. Recent years have witnessed a surge of interest in *VMA21*, with the identification of novel mutations causing a congenital disorder of glycosylation (CDG) with liver affection, and its potent implication in cancer predisposition. With this, VMA21 deficiency has been further linked to defective glycosylation, lipid metabolism dysregulation and ER stress. Moreover, the identification of two VMA21 isoforms, namely VMA21-101 and VMA21-120, has opened novel avenues regarding the pathomechanisms leading to XMEA and *VMA21*-CDG. In this review, we discuss recent advances on the clinical spectrum associated with VMA21 deficiency and on the pathophysiological roles of VMA21.

## Introduction

During the past decades, genetic studies in humans have increasingly linked insufficient lysosomal acidification to pathological conditions, such as neurodegenerative disorders, cancer, inflammatory diseases and myopathies.^1–4^ Impaired lysosomal acidification results in the accumulation of toxic substrates by disrupting autophagy, a catabolic process engulfing and ultimately degrading cytoplasmic components and organelles.^5^ Lysosome acidification, sustaining the hydrolytic activity of luminal enzymes, involves the vacuolar ATPase (v-ATPase) proton pomp at the lysosomal membrane. Assembly of the two large domains forming the v-ATPase occurs sequentially in the endoplasmic reticulum (ER) and Golgi, and is controlled by chaperone proteins, such as TMEM199, CCDC115 and VMA21. Mutations in genes encoding sub-units or chaperones of the v-ATPase cause various diseases, associated with lysosomal acidification impairment.^6^ In particular, mutations in the *VMA21* gene has been identified in 2013 as the genetic cause of a rare neuromuscular disorder, called X-linked Myopathy with Excessive Autophagy (XMEA),^7^ and several years later of a congenital disorder of glycosylation (CDG) affecting the liver.^8^ More recently, *VMA21* variants were associated with follicular lymphoma (FL), highlighting for the first time the role of VMA21 in cancer development.^9^ Impaired autophagy, consistent with a mis-assembly of the v-ATPase, has been incriminated as one of the key pathomechanisms of *VMA21*-related diseases. However, autophagy defect cannot solely explain the heterogeneous clinical spectrum and the tissue specificity observed in these diseases. The recent discovery of distinct VMA21 isoforms also raises questions on their respective pathophysiological roles in the disorders. In this review, we dissect the clinical features of XMEA, CDG and FL, and we compare the pathomechanisms that may contribute to the pathogenesis of these diseases. Moreover, we provide a complete list of the mutations reported in the three conditions, and discuss their functional consequences and the associated clinical diversity.

## *VMA21*-related disorders: from genetic basis to clinical diversity

### XMEA: a rare autophagic vacuolar myopathies

#### Autophagic vacuolar myopathies (AVMs)

AVMs constitute a group of neuromuscular diseases characterized by autophagic vacuoles in skeletal muscle biopsies from patients. These vacuoles accumulate in muscle fibres and stain positive for autophagic and lysosomal protein markers. They commonly originate from a dysregulation of the lysosome-autophagy process, although the pathomechanisms differ from one disease of the family to another.^10^

A first group of AVMs is caused by primary lysosomal dysfunction. The two most prevalent AVMs of this group are Pompe disease (or glycogen storage disease II, GSDII) and Danon disease (or glycogen storage disease IIb, GSDIIb). Pompe disease is caused by mutations in the *GAA* gene, which encodes the lysosomal enzyme acid alpha-glucosidase (GAA), essential for the degradation of glycogen to glucose. In Pompe disease, GAA deficiency results in the accumulation of glycogen in lysosomes, which impairs the autophagy process and lead to the formation of large autophagic vacuoles.^11^ Severe forms of Pompe disease manifest at birth with cardiomyopathy and muscle weakness, while late-onset forms are characterized by progressive motor dysfunction and respiratory insufficiency. While Pompe disease arises from the deficiency in a unique lysosomal enzyme, Danon disease is caused by mutations in the *LAMP2* (lysosomal associated membrane protein 2) gene on the X chromosome, which encodes a glycoprotein essential for lysosomal integrity and functioning.^12^ Male patients typically present with a multisystemic phenotype, including cardiomyopathy, muscle weakness, and intellectual disability. Female patients usually exhibit a milder and delayed phenotype, primarily featuring cardiomyopathy. Similar to Pompe disease, glycogen accumulates in skeletal muscle in Danon disease. Consequently, both Danon and Pompe diseases also belong to the large group of lysosomal storage disorders (LSDs), characterized by deficiencies in proteins crucial for lysosomal function and the accumulation of specific materials within lysosomes.^13^ Of note, autophagic vacuoles in Danon disease stain positive for (sub-) sarcolemmal components. This unusual nature of the vacuoles inspired Nishino et al. to introduce the term “autophagic vacuoles with sarcolemmal features” (AVSF).^14,15^ XMEA is another AVM involving primary lysosomal dysfunction that will be discussed in detail in the next parts, in terms of clinical, histological and pathological features.

The other group of AVMs is distinguished by the presence of rimmed vacuoles and includes sporadic inclusion body myositis, GNE myopathy and myofibrillar myopathies.^16–18^ These myopathies are primarily caused by extra-lysosomal alterations, suggesting that the accumulation of rimmed vacuoles results from secondary lysosomal dysfunction.

#### XMEA: clinical aspects

The first description of XMEA in 1988 provided clinical reports of five males with X-linked inheritance and weakness of proximal limb muscles.^19^ The disease had a very slow progression, with an onset at early childhood. The patients exhibited difficulties in standing up from a sitting position, but they all retained ambulation ability. Creatine kinase (CK) was elevated and no organ systems other than skeletal muscle was affected. In 1995, a second report similarly described five male patients with a juvenile onset, predominant proximal muscle involvement and slow progression of the disease.^20^ In the following years, other XMEA cases were reported with equivalent clinical features.^7,21–30^ Some patients showed high frequency discharges during electromyography without typical symptoms of myotonia.^31^ Magnetic resonance also revealed muscle atrophy and degeneration, with fat replacement in several patients.^21,22,26,28,31–33^ There is generally no sign of cardiac or intellectual involvement in XMEA patients.^34^ Notwithstanding, cardiac hypertrophy and vacuolation were reported in one 27-month-old infant, who died from respiratory insufficiency.^35^ In parallel, one XMEA patient^7^ experienced fatal hepatic cirrhosis at 52 years of age.^36^ Liver biopsy revealed vacuolation pattern, suggesting that XMEA pathology is not solely confined to skeletal muscles. The authors proposed that other factors had caused his liver disease, which was ultimately accelerated by *VMA21* deficiency.^36^ Finally, two XMEA cases were reported with elevated urinary β2 microglobulin, although with no evident renal dysfunction.^21^

Strikingly, the age of onset and the degree of severity considerably vary between cases, even in between individuals from the same family.^37^ The first reported case of late adult-onset was a 65-year-old patient, with symptoms observed around the age of 55.^38^ This patient displayed slowly progressive proximal lower limb weakness and ambulation supported by mechanical assistance. At the opposite extreme of the clinical spectrum, severe forms of XMEA associate with neonatal onset. Symptoms include early ambulation loss, as well as severe proximal weakness of upper and lower limb muscles.^32,39–42^ A few patients also exhibited impaired ocular movements.^23,25,39,41^ Five male infants died in the neonatal period due to their inability to breathe and suckle. Two other family members survived through nasogastric feeding and intubation, and displayed typical XMEA features.^43^ In another family, two brothers died at 7 and 17, due to multiorgan and heart failure, respectively; their cousin died at birth because of respiratory failure, while their nephew showed extremely severe XMEA.^41^ Notably, two twin female patients reported with progressive myopathy may be cases of carrier females clinically affected by XMEA, although there was no genetic confirmation of this diagnostic.^44^ The variability observed in XMEA is commonly observed in inherited diseases and may be attributed to genetic or environmental modifiers. However, the small number of XMEA patients reported so far makes it challenging to identify contributing factors. Despite the relatively unspecific clinical presentation of XMEA, the systematic histological characterization of skeletal muscle biopsies, followed by genetic analyses, has greatly accelerated XMEA diagnosis in the last years. Thanks to a prenatal testing approach, a case of XMEA was also diagnosed in a male foetus displaying micrognathia, short limbs, and arthrogryposis.^45^ In the mother's previous pregnancy, the foetus showed comparable anomalies and died shortly after birth, which may indicate another case of neonatal lethality due to *VMA21* mutations.

#### XMEA: histopathological features

Histopathological features associated with XMEA are homogeneous between patients. Muscle biopsies typically show myopathic features, with variation in fibre size, muscle fibre splitting, and infrequent necrosis. As in other AVMs, they display autophagic vacuoles in muscle fibres. These cytoplasmic vacuoles generally stain positive for sarcolemma-associated proteins, such as dystrophin, caveolin-3 or dysferlin, and for some basal lamina proteins (*e.g.,* laminin-α2, perlecan), leading to the denomination AVSF, as in Danon disease.^46,47^ Immunostaining for lysosomal proteins, such as LAMP2, reveals accumulation of lysosomes in XMEA biopsies. Positive staining for LAMP2 distinguishes XMEA from Danon disease, marked by LAMP2 loss, and is used as a key diagnostic element. The presence of small to large vacuoles with single or double membranes in XMEA muscle biopsies can be confirmed by electron microscopy.^46,47^ In some cases, autophagic vesicles are located close to the sarcolemma, suggesting extrusion of their contents. In these areas, the basal lamina appears duplicated.^19,21,23–26,38–40,42,43^ Finally, inflammatory markers, such as membrane attack complex with C5b-9 deposition or major histocompatibility complex class I, were reported at the membrane of muscle fibres, as well as within the cytoplasmic vacuoles.^20,48–50^ Muscle biopsies also show calcium deposition within some fibres and along the sarcolemma.^51^

### XMEA: identification of the genetic cause

The first description of XMEA families in 1988 and 1995 clearly linked the disease to the X chromosome.^19,20^ Linkage analysis based on polymorphic DNA markers narrowed down the region co-segregating with the disease phenotype to the segment Xq28.^52–54^ Follow-up studies on the inheritance of highly polymorphic micro-satellites (or short tandem repeats) present in the Xq28 region in XMEA families further narrowed down the number of putative causative genes.^55,56^ In 2013, whole-exome sequencing applied to several affected individuals from XMEA families led to the identification of pathogenic mutations in the *VMA21* gene, present in the Xq28 region.^7^ Since this report, several other patients diagnosed with XMEA have been reported with mutation identified in *VMA21*.^21,22,24,25,28,30,32,33,35,38,39,41,43^ A systematic review of all XMEA mutations is shown in Table 1. The table also gives information about the number of patients, disease onset, severity, and clinical manifestations associated with each mutation.

**Table 1.** Clinical patterns in XMEA and CDG patients affected by *VMA21 *mutations.

Mutations	#Patients	#Families	Onset^a^	Severity	Clinical features^b^	Ref.
c.-10C > T (5′UTR)	1	1	?	Mild	Steatosis	^8^
c.52A > G (Ex1-101)	1	1	?	Mild	Steatosis and mild hypercholesterolemia	^8^
c.54-30_54-27delinsT (Intron1)	5	1	Childhood	Mild	Calves pain, lower limb weakness	^33^
c.54-27A > C (Intron1)	5	3	Childhood	Mild	Predominant proximal lower limb weakness and extraocular muscle paralysis	^7,25^
c.54-27A > T (Intron1)	1	1	Childhood	Mild	Predominant proximal lower limb weakness	^7,24^
c.54-16_54-8del (Intron1)	1	1	Neonatal	Severe	Early ambulation loss, proximal and distal muscle weakness	^39^
c.94_96delTTC (Exon2)	1	1	Foetal	Severe	Foetal arthrogryposis and micrognathia	^45^
c.163 + 3A > G (Intron2)	2	1	Childhood	Mild	Predominant proximal lower limb weakness	^28^
c.163 + 4A > G (Intron2)	8	5	Childhood	Mild	Proximal and distal lower limb weakness	^7,26,28^
c.164-20T > A (Intron2)	1	1	Childhood	Mild	Strong proximal weakness, myotonic discharges	^32^
c.164-7T > G (Intron2)	15	5	Childhood or late-adult	Mild	Predominant proximal lower limb weakness	^7,21,38^
c.164-7T > A (Intron2)	1	1	Early adulthood	Mild	Proximal lower limb weakness	^24^
c.164-6T > G (Intron2)	7	1	Congenital	Severe	Muscle wasting, scoliosis, mild cardiac hypertrophy and respiratory failure	^43^
c.188A > G (Exon3)	1	1	Childhood	Mild	Steatosis and mild hypercholesterolemia	^8^
c.272G > C (Exon3)	13	4	Childhood or adulthood	Mild	Predominant proximal lower limb weakness	^7,22,26^
c.294C > T (Exon3)	4	1	Congenital	Severe	No ambulation, permanent mechanical ventilation, multiorgan failure	^41^
c.*6A > G (3’UTR)	20	3	Childhood	Mild	Predominant proximal lower limb weakness	^7,36^
c.*13-*104del (3’UTR)	1	1	Neonatal	Severe	Early ambulation loss, proximal and distal muscle weakness	^39^

^a^ Different onsets correspond to the same mutation but different patients ; ^b^Different clinical symptoms for a given mutation correspond to different patients. Grey and white rows correspond to XMEA and CDG patients, respectively.

**Figure 1.**
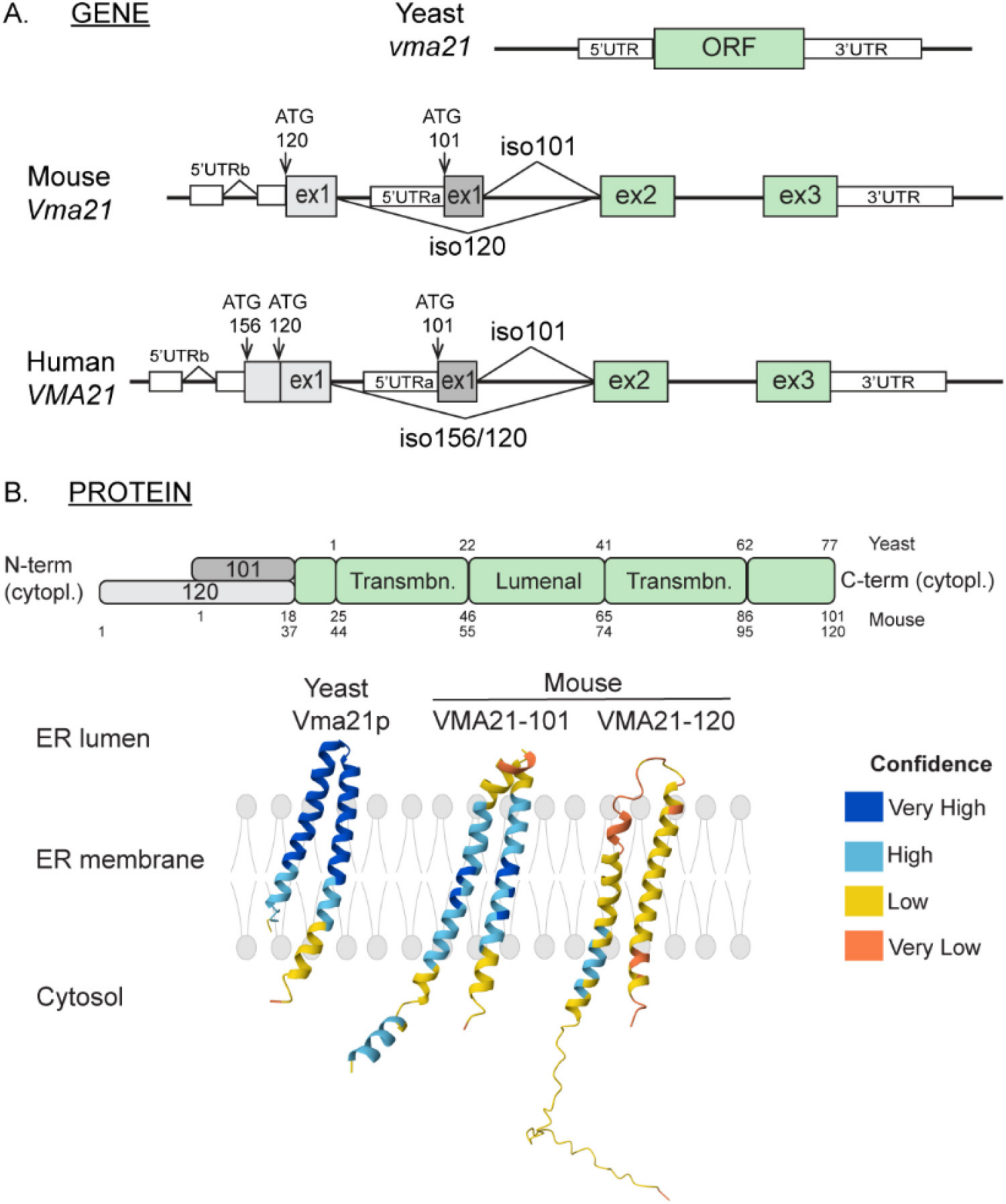
VMA21: from gene to protein. A. Organization of the *VMA21* gene in yeast, mouse and human. Exons and untranslated (UTR) regions are represented by grey (exon 1), green (exons 2/3) and white boxes (UTR). Alternative ATG codons are indicated with arrows. B. Representation of VMA21 protein structure in yeast and mouse. Protein topology prediction for VMA21 proteins were obtained with AlphaFold.^57,58^ *Cytopl : cytoplasmic ; Transmbn : transmembrane.*

In the following parts, the nomenclature for the mutations refers to the *VMA21-101* transcript (NM_001017980.4), in line with previous case reports. Mutations identified so far are single nucleotide substitutions and microdeletions found mainly in intronic regions of *VMA21* (see Figure 1 for the organization of the gene). Classical forms of XMEA were associated with the substitutions c.54-27A > T, c.54-27A > C, c.163 + 3A > G, c.163 + 4A > G, c.164-7T > G, c.164-7T > A and c.272G > C.^7,21,22,24,25,28,30^ The late onset form of XMEA was also associated with the mutation c.164-7T > G, suggesting involvement of genetic modifiers or environmental factors.^38^ Congenital and neonatal cases involved the microdeletions c.54-16_54-8del in the intron 1 and c*.13_*104del in the 3′UTR region, as well as the substitutions c.164-6T > G and c.294C > T.^39,41,43^ Of note, the mutation c.164-6T > G is located at one nucleotide from the c.164-7T > G mutation associated with classical forms. The patient with cardiac involvement displayed the same mutation (c.164-6T > G),^35^ while the XMEA patient with hepatic failure carried a mutation in the 3′UTR region (c.*6A > G).^36^ Finally, the prenatal case was associated with the non-frameshift deletion mutation c.94_96delTTC located in a highly conserved region of VMA21.^45^ The functional consequences of these mutations will be discussed afterwards.

### Congenital disorder of glycosylation: expanding the clinical spectrum of *VMA21*-related disorders

In 2020, mutations in the *VMA21* gene were shown to cause a CDG marked by liver disease.^8^ CDGs are a group of multisystemic disorders characterized by alterations in the synthesis of glycans.^59,60^ They are classified based on the type of glycosylation that is altered, *e.g.,* protein N- or O-glycosylation, or glycosphingolipid synthesis. CDGs are caused by an impaired activity and/or expression of enzymes involved in glycosylation, such as glycosidases or glycotransferases, or by an altered activation and/or transport of sugar precursors. The origin of the disease determines the type of glycosylation defect and the clinical pattern observed in the patients. In most CDGs, the liver is clinically affected, contributing to the multisystemic phenotype observed in these patients.^61^ Some CDG patients display neurological abnormalities, including mental retardation, seizures, and/or microcephaly.^62^ Few patients exhibit cardiac involvement.^63^ Screening tests for CDGs are based on the analysis of glycoprotein separation (isoelectric focusing, IEF) based on their isoelectric point. This typically includes transferrin, a predominant glycoprotein in the serum that contains 2 sites for N-glycans eventually terminated with sialic acid. Defects in N-glycan synthesis result in the improper incorporation of sialic acid, which is detectable by IEF in the serum of patients with CDGs.^64,65^ More than 100 genes have been shown to cause CDGs, with an alteration of the N-glycosylation in the majority of these diseases.

Three male patients with *VMA21* mutations have been diagnosed so far with CDG syndrome (Table 1).^8^ Abnormal N- and O-glycosylation of plasma proteins was documented for all patients, with the accumulation of markers, such as transferrin or ApoCIII, with low sialylation. The three patients exhibit clinical features ranging from defective bile flow from liver (cholestasis) and fat retention in liver (steatosis) to mild hypercholesterolemia. These symptoms were associated with increased levels of alanine aminotransferase (ALT) and aspartate aminotransferase (AST) detected at early ages, followed by hypercholesterolemia for two of these patients.^8^ There was no sign of myopathy reported for these patients. Of note, levels of ALT/AST tested in one XMEA patient in this study were normal, while cholesterol levels were increased.^8^ Ultrastructural analysis of liver biopsies from *VMA21*-CDG patients revealed mild steatosis and irregularly shaped hepatocytes, without inflammation or fibrosis. Hepatocytes displayed accumulation of lipid droplets within lysosome-related vesicles, multivesicular bodies, as well as dilated Golgi. The mutations identified in *VMA21*-CDG patients are single nucleotide substitutions detected before the exon 1 (c.-10C > T), in the exon 1 (c.52A > G) and in the exon 3 (c.188A > G).^8^ These mutations were not previously reported in XMEA patients, which may explain, at least in part, the clinical heterogeneity observed between patients. Notably, mutations in the genes *ATP6AP1*, *ATP6AP2*, *TMEM199* and *CCDC115*, encoding the four other assembly chaperones of the v-ATPase, also cause CDGs with a heterogeneous clinical spectrum including hepatic injury and steatosis.^66–70^ The corresponding reports do not document myopathic syndrome for these patients. However, some of them presented with psychomotor impairment, hypotonia or muscle weakness, indicating potential skeletal muscle involvement.^66–69^

### Cancer: a potential role of VMA21?

#### Follicular lymphoma (FL)

FL represents a common indolent B-cells lymphoma with an estimation of 100.000 cases in the US in 2016.^71^ Its incidence strongly varies depending on ethnicity and geography, and it tends to increase with age. Lymphoma usually involves bone marrow and lymph nodes. Symptoms of FL can be subtle and develop gradually, with usually swelling of lymph nodes in different regions of the body, such as the neck, the abdomen, and more rarely, the inguinal region.^72^ Around 20% of the patients also experiences fatigue, fever, night sweats and weight loss. Most FL patients carry the somatic chromosome translocation t(14;18) (q32;q21), which results in the constitutive activation of B-cell lymphoma 2 (BCL2) oncogenic protein.^73^ In parallel, 10% of FL cases are associated with mutations in the gene *RRAGC* encoding the small G-protein Ras Related GTP Binding C (RagC), a component of the complex Ragulator that signals amino acid levels and mediates the activation of mammalian Target of Rapamycin Complex 1 (mTORC1). Consistently, increased mTORC1 activity is central in FL and constitutes a potential therapeutic target.^74^ More recently, hotspot mutations in the gene *ATP6V1B2* encoding a v-ATPase subunit were also associated with LF and mTORC1 hyperactivation.^9^ Exome sequencing of FL B-cells led to the identification of other mutated genes, including *ATP6AP1*, *ATP6AP2* and *VMA21.*^75^ Of note, *VMA21* mutations were present in cells with concurrent mutations in *KMT2D, EP300* and *CREBBP*, suggesting that *VMA21* contributes as a modifier factor in the development or progression of FL.^9^ The hotspot mutation identified in *VMA21* in FL cells corresponds to the nonsense substitution c.277C > T, which has been reported neither in CDG nor in XMEA. The mutation results in the deletion of nine amino acids in the C-terminal region of VMA21 (p.R93X).^9^ Other nonsense mutations (c.263G > A and c.284G > A) affecting the C terminus, as well as several missense mutations (c.128G > A, c.142A > C, c.54-1G > A) were identified in LF cells. Expression of the mutant forms of VMA21 would give a selective advantage to the mutated FL B-cells (see the pathomechanisms discussed below).

#### VMA21 expression and cancer prognosis

**Figure 2.**
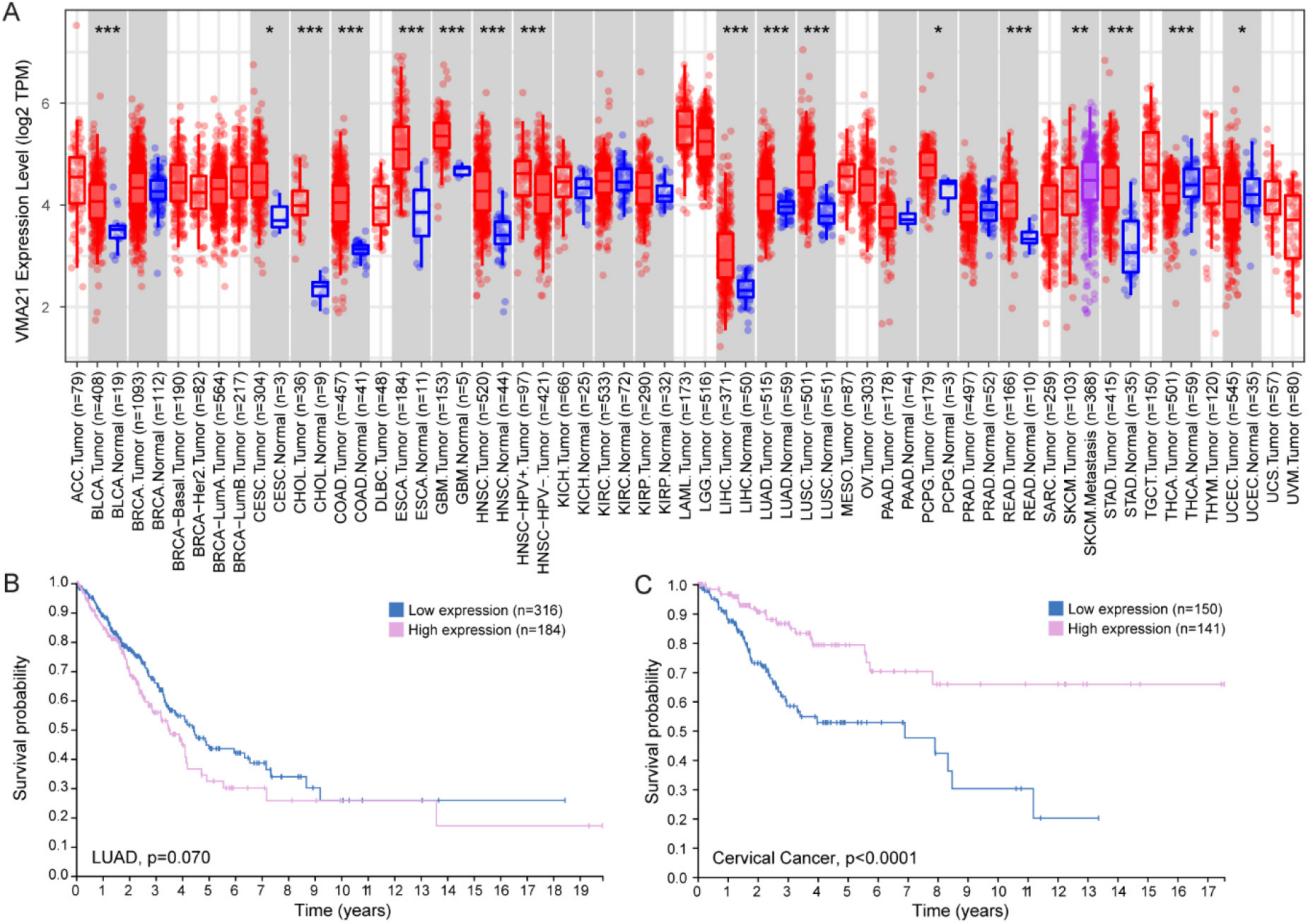
VMA21 in cancer. A. Expression pattern of *VMA21* in tumour and normal tissues obtained from TIMER2.0.^76^ Distribution of gene expression levels are displayed using box plots. Wilcoxon test, * p < 0.05, ** p < 0.01, *** p < 0.001. ACC : adrenocortical carcinoma; BLCA : bladder urothelial carcinoma; BRCA : breast invasive carcinoma; CESC : cervical and endocervical cancer; CHOL : cholangiocarcinoma; COAD : colon adenocarcinoma; DLBC : lymphoid neoplasm diffuse large B-cell lymphoma; ESCA : oesophageal carcinoma; GMB : glioblastoma multiforme; HNSC : head and neck cancer; KICH : kidney chromophobe; KIRC: kidney renal clear cell carcinoma; KIRP : kidney renal papillary cell carcinoma; LAML : acute myeloid leukaemia; LGG : brain lower grade glioma; LIHC : liver hepatocellular carcinoma; LUAD : lung adenocarcinoma; LUSC : lung squamous cell carcinoma; MESO : mesothelioma; OV : ovarian serous cystadenocarcinoma; PAAD : pancreatic adenocarcinoma; PCPG : pheochromocytoma and paraganglioma; PRAD : prostate adenocarcinoma; READ : rectum adenocarcinoma and stomach; SARC : sarcoma; SKCM : skin cutaneous melanoma; STAD : stomach adenocarcinoma; TGCT : testicular germ cell tumors; THCA : thyroid carcinoma; THYM: thymoma; UCEC : uterus corpus endometrial carcinoma; UC : uterine carcinosarcoma; UVM : uveal melanoma. B, C. Survival analysis of 490 LUAD patients and 291 cervical cancer patients, with low or high *VMA21* expression levels. Data are from The Human Protein Atlas.^82^

The TIMER2.0 database^76^ suggests changes in *VMA21* transcript levels in several cancer types, as compared to healthy cells (Figure 2A). In line with this, up-regulation of *VMA21* expression was confirmed in lung adenocarcinoma (LUAD) cell lines, as compared to normal human bronchial epithelial cells.^77,78^ This was linked to increased levels of the long non-coding RNA (lncRNA) *ZFPM2-AS1* and of the circular RNA *circRNA-0001361* in LUAD cells, which sponged the inhibitory effect of the microRNAs miR-18b-5p and miR-525-5p towards *VMA21* transcript.^77,78^ Similarly, VMA21 transcript and/or protein levels were increased in ovarian cancer cells, melanoma cell lines and cervical cancer cells, which was associated with the up-regulation of other specific lncRNAs.^79–81^ These changes suggest that VMA21 may play a role in cancer onset and/or progression. This is supported by the correlation observed between VMA21 expression and the prognosis of certain cancers. As such, a positive correlation between VMA21 expression and survival has been reported for colorectal cancer (CRC) . In contrast, high VMA21 expression was linked to poor prognosis in ovarian cancer, melanoma, LUAD (Figure 2B – not significant in the Human Protein Atlas (THPA) database^82^) and cervical cancer.^78–81^ High VMA21 expression mediated the effect on cell growth of lncRNAs specifically up-regulated in each cancer type (*e.g., LOXL1-AS1* in ovarian cancer).^78–81^ VMA21 up-regulation may thus be a strong contributor to tumorigenesis in these cancers. Of note, the THPA database indicates that high VMA21 levels rather correlate with favourable patient outcome in cervical cancer (Figure 2C). Finally, the International Cancer Genome Consortium documents the single base substitution c.277C > T (p.R93X) identified in FL with high functional impact in cancers. This variant has been found in one donor with oesophageal cancer and one with colon adenocarcinoma over 332 and 402 donors, respectively. Overall, VMA21 deregulation may associate with either a favourable or unfavourable prognosis depending on the cancer type. This dual effect may be driven by cell type-specific signalling pathways related to cell growth and death, and distinct sensitivities and dependencies to autophagic flux.

## VMA21: from gene to cellular (dys)functions

### From gene to protein

#### VMA21: gene, transcripts and protein isoforms

The *Vma21* gene was first discovered in 1994 in *Saccharomyces cerevisiae* by K. J. Hill and T. H. Stevens from studies screening mutants deficient for v-ATPase function.^83^ In yeast, the *Vma21* gene is composed of one exon and codes for a small protein of 77 amino acids. Remarkably, *Vma21* null mutants exhibit defective vacuole acidification and undetectable v-ATPase activity, together with impaired growth.^83^ In mammals, the *VMA21* gene is located on the X chromosome (Xq28 region in human) and the coding sequence is composed of three exons. Different *VMA21* transcripts are predicted *in silico* in human, as well as in other mammals, such as mice. These transcripts arise from alternative transcription start sites (TSS) and share exons 2 and 3, but not exon 1 (Figure 1A). We recently demonstrated that two main transcripts are expressed in humans and mice: one short transcript (*VMA21-101*) reported in prior studies and one longer transcript (*VMA21-120*).^84^ Notably, a third transcript, *VMA21-156,* was detected in human but not in mice. The TSS of *VMA21-120/156* are located upstream the TSS of *VMA21-101* (Figure 1A).

In yeast, *Vma21* encodes Vma21p of 8.5 kDa. In human, the alternative transcripts encode a 101-, 120- and 156-amino acid long proteins, of 11.5, 13.5 and 18 kDa, which differ in their N-terminal regions^84^ (Figure 1B). In both human and mouse, VMA21-101 expression is detected in most tissues. In contrast, VMA21-120 expression is restricted to skeletal muscles and is higher during muscle development and regeneration in mice. Consistently, VMA21-120 expression strongly increases after inducing muscle cell differentiation *in vitro*, before the fusion of committed muscle precursors.^84^ Based on RNAseq data, *VMA21-120/156* also seem to be specifically expressed in the muscle lineage in human.^84^ Low levels of these isoforms were found in heart. Protein sequence alignment revealed a strong conservation throughout species for the region shared by the three isoforms (central and C-term). The N-terminal region of human VMA21-101 shows high similarities with homologous proteins found in rodents, fish or worm, but not in *S. cerevisiae.*^84^ Notwithstanding, expression of human VMA21-101 was sufficient to reverse growth defects in yeast *vma21* null mutants.^7^ Homologs of VMA21-120 were identified only in mammals, and only in primates for VMA21-156.^84^ VMA21 isoforms have also been described in plants: AtVMA21a and AtVMA21b share around 28% protein similarity with Vma21p, and were both sufficient to compensate for Vma21p depletion in yeast.^85^

#### From *VMA21* mutations to clinical spectrum

**Figure 3.**
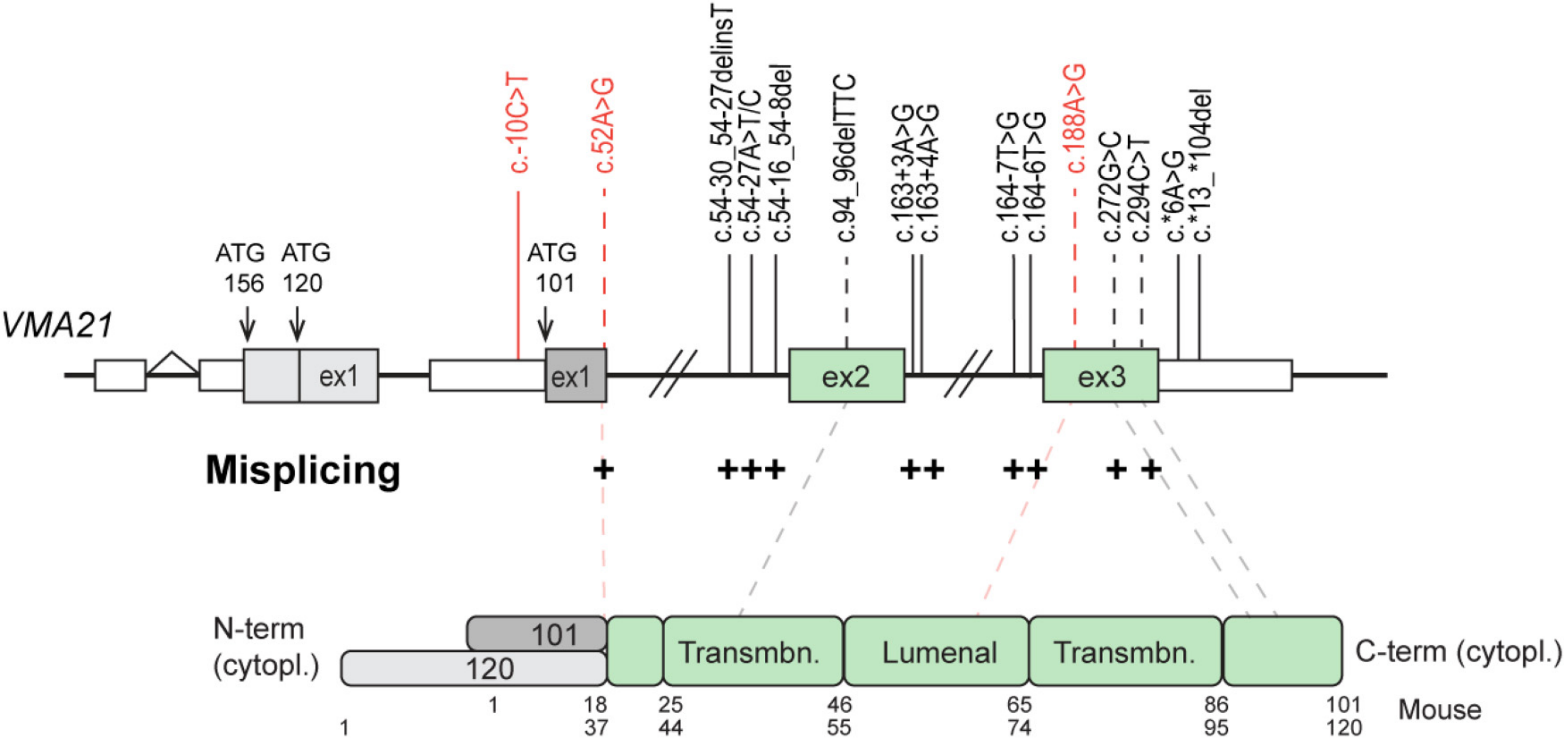
Spectrum of *VMA21* mutations identified in CDG and XMEA. Mutations identified in XMEA and CDG patients are in black and red, respectively. The position of the TSS of the three *VMA21* isoforms are shown with arrows on the gene (top). Exons and UTR regions are shown with boxes; introns are depicted with bridging gaps and lines. Amino acids numbers are indicated below the protein domains (down). Mutations in coding region are positioned at the protein with dotted lines. Mutations affecting splicing are indicated with + . *Cytopl: cytoplasmic; Transmbn: transmembrane.*

Mutations found in patients affected by XMEA or CDG are distributed across the entire *VMA21* gene (Figure 3), with the exception of *VMA21-120* exon 1 and surrounding regions, which have not been, to our knowledge, included in the exome strategy sequencing so far. All *VMA21* mutations are inherited in a recessive X-linked manner and correspond to microdeletions or single base substitutions. The outcome of *VMA21* mutations is illustrated in Table 2. As discussed above, most mutations associated with XMEA are in intronic regions, affecting donor or acceptor splicing sites. In particular, Ramachandran and al. (2013) initially described 4 substitutions in non-coding regions with distinct effects on RNA splicing: the mutations c.54-27A > T and c.54-27A > C result in loss of the adenine critical for the splice branch point of intron 1; c.163 + 4A > G eliminates the adenine in the fourth position after exon 2, impairing the binding of the U1 snRNA during splicing; and c.164-7T > G before the exon 3 is proposed to decrease U2AF splice factor binding efficiency.^7^ Notably, the mutation c.272G > C in the coding sequence (p.Gly91Ala) and the silent mutation c.294C > T (p.Gly98=) would also affect *VMA21* splicing.^41^ In parallel, mutations reported in the 3′UTR region (c.*6A > G or c.*13_*104del) in proximity to the stop codon, were associated with transcript destabilization.^7,39,84^

**Table 2.** Effect of *VMA21* mutations on mRNA and protein levels of VMA21.

*VMA21* mutation	Consequences	mRNA levels (% of ctrl)	Protein levels (% of ctrl)^a^	Cell type studied
c.-10C > T (5’UTR)	Alternative ATG: premature stop codon	≈25%	≈12.5%	Fibroblasts
c.52A > G (Exon1-101)	Perturbs 5’ splice site, mis-splicing and premature stop codon; Missense mutation, p.Arg18Gly	≈25%	≈12.5%	Fibroblasts
c.54-30_54-27delinsT (Intron1)	Disrupts branch point in intron 1	≈10%	NR	Muscle cells
c.54-27A > C (Intron1)	Disrupts branch point in intron 1	NR	NR	Lymphoblasts
c.54-27A > T (Intron1)	Disrupts branch point in intron 1	≈50%	≈30-40%	Lymphoblasts
c.54-16_54-8del (Intron1)	Disrupts 3’ splice site	≈40-50%	Undetected	Lymphoblasts
c.94_96delTTC (Exon2)	Deletion p.Phe32del	NR	NR	Prenatal diagnosis
c.163 + 3A > G (Intron2)	Disrupts 5'splice site and binding of U1 snRNA	NR	NR	
c.163 + 4A > G (Intron2)	Disrupts 5’ splice site and binding of U1 snRNA	≈55%	NR	Lymphoblasts
c.164-20T > A (Intron2)	Disrupts 3’ splice site; mis-splicing	≈40%	Undetected	Fibroblasts
c.164-7T > G (Intron2)	Disrupts 3’ splice site and binding of U2AF splice factor	≈60%	Strongly reduced	Lymphoblasts/Muscle biopsy
c.164-7T > A (Intron2)	Disrupts 3’ splice site and binding of U2AF splice factor	NR	NR	
c.164-6T > G (Intron2)	Disrupts 3’ splice site, binding of U2AF splice factor	≈25%	NR	Lymphoblasts
c.188A > G (Exon3)	Missense mutation, p.Asp63Gly	≈50%	≈25%	Fibroblasts
c.272G > C (Exon3)	Disrupts SC35 splice factor binding; Missense mutation, p.Gly91Ala	≈70%	≈30-40%	Lymphoblasts
c.277C > T (Exon3)	Nonsense mutation, p.R93X; Mislocalization	NR	NR	Follicular Lymphoma
c.294C > T (Exon3)	Silent mutation, p.Gly98; Mis-splicing	NR	NR	
c.*6A > G (3’UTR)	Disrupts 3’UTR and binding of processing factors	≈45%	≈30-40%	Lymphoblasts
c.*13-*104del (3’UTR)	Disrupts 3’UTR and binding of processing factors	≈40-50%	Undetected	Lymphoblasts

^a^ Protein levels evaluated by Western Blot. NR: *not reported*. Dark grey, light grey and white rows correspond to mutations identified in FL, XMEA and CDG, respectively. The references for each mutation are given in Table 1.

The severity of XMEA has been linked to the residual levels of VMA21. Indeed, severe cases showed drastic reduction in VMA21 transcript or protein levels in lymphoblasts or fibroblasts, while 30-60% remaining expression levels were reported for other XMEA mutations (Table 2). However, VMA21 protein levels have not been evaluated for all mutations. Moreover, recent studies reported lower transcript and/or protein levels when using muscle cells.^33,84^ Hence, the consequences of each mutation on VMA21 expression, especially focusing on both VMA21 isoforms, should be evaluated in muscle cells, to get a more representative view of VMA21 deficiency in XMEA patients, and of the correlation between VMA21 levels and disease severity.

Analysis of *VMA21* mutations in CDG patients by Cannata Serio et al. (2020) also provided information about their molecular consequences.^8^ The mutation c.-10C > T in the 5′ UTR of *VMA21-101* produces a new ATG initiation codon located 11 bp upstream of *VMA21-101* exon 1, which results in a premature stop codon in between nucleotides 26–28 of *VMA21* coding sequence. Similarly, the mutation c.52A > G in exon 1 of *VMA21-101* leads to the formation of a predominant alternative splice variant and to a premature stop codon immediately at the end of the exon 1. Fibroblasts from CDG patients carrying these mutations expressed only 10-20% remaining VMA21 protein levels. Finally, the mutation c.188A > G in exon 3 results in the substitution of an asparagine to glycine (p.Asp63Gly) in the luminal domain, with around 30% reduction in protein levels compared to control.^8^ Whether the function of the mutated protein is preserved remains unknown.

Overall, it is difficult to explain the distinct clinical spectrum between XMEA and CDG on the basis of the remaining expression levels of VMA21. In most studies, only the levels of VMA21-101 have been evaluated in cells from patients. The microdeletion c.54-30_54-27delinsT in intron 1, reported in a Brazilian XMEA patient, strongly alters the expression of both VMA21 isoforms.^33,84^ The mutation led to an alternative *VMA21-120* transcript containing a small sequence of intron 1, which resulted in a premature stop codon; the corresponding protein was undetected in patient cells. Similarly, the mutation c*13_*104del, reported in an Italian XMEA patient, reduces by 70-80% *VMA21-101* and *VMA21-120* transcript levels in patient myotubes.^39,84^ The specific expression of VMA21-120 in skeletal muscle, as well as the localization of *VMA21* mutations in XMEA vs. CDG patients, suggest that the loss of VMA21-120 may contribute to XMEA pathogenesis. Mutations affecting VMA21-120 expression may primarily trigger muscle phenotypes. Consistently, all XMEA patients carry mutations close to or in exon 2, exon 3 or in the 3′UTR, which are common sequences of *VMA21-101* and *-120* transcripts. In contrast, two of the CDG mutations were found in or close from *VMA21-101* exon 1, which may preserve VMA21-120 expression and spare skeletal muscles in CDG patients (Figure 3). However, why the loss of VMA21-101 in XMEA patients does not result in liver or other non-muscle tissue alterations is still unknown. Further investigation on the expression levels of *VMA21-120* in XMEA and CDG, as well as inclusion of *VMA21-120* exon 1 in exome sequencing strategy, will be decisive in better understanding VMA21-associated disorders.

### VMA21: properties of the chaperone protein

Sequence analysis and Alpha Fold^57,58^ predict that VMA21 is a transmembrane protein with both N- and C-terminal regions exposed to the cytosol^83^ (Figure 1B). CryoEM in yeast confirmed the α helical hairpin topology of Vma21p within the membrane, with the two side chains in the cytosol.^86^ AlphaFold predictions suggest differences between VMA21 isoforms in their respective N-terminal domain. The N-terminal region of VMA21-120 consists of 37 residues, which would form a flexible domain in the cytoplasm. In contrast, VMA21-101 would feature a short helical domain (residues 2-11), followed by a 3-residue linker (residues 12-14) connected to the first transmembrane helix (Figure 1B). The different length and conformation of the N-terminal domains of VMA21 isoforms may impact the structural dynamics, the interactions with other proteins, and thereby the function of each isoform.

The topology of Vma21p/VMA21 is consistent with its localization in the membrane of organelles. Most studies have revealed an accumulation of the protein in the ER in yeast^83,87^ and in mammals.^7,84^ The protein was also found in the ER-Golgi intermediate compartment (ERGIC) in muscle cells and lymphoblasts.^7,84^ In contrast, VMA21 was undetectable in lysosomes or in the Golgi. In yeast, Vma21p contains a carboxy-terminal di-lysine motif (KKXX) that is critical for its ER retention. Mutagenesis of these lysine residues resulted in Vma21p delivery to the yeast vacuolar membrane.^83^ The di-lysine motif is conserved in plants, but not in animals. The alternative motif contains a single lysine and an acidic aspartate residue (XKXD), which would ensure the retention and traffic back of VMA21 to the ER.^7,9^ Indeed, mutation of the KQD motif of the human VMA21 led to its mislocalization to lysosomes, providing evidence that the KQD residues serve as an atypical ER retrieval signal.^9^

### VMA21: an assembly chaperone of the v-ATPase

#### Functions and organization of the v-ATPase

v-ATPases are large protein complexes found in all eukaryotes, which form membranous proton pumps, essential for the acidification of organelles and intracellular vesicles. This acidification sustains specific functions in sub-cellular compartments.^88^ In lysosomes, the low pH (pH 4-5) confers an optimal environment for the activity of degradative enzymes. In Golgi, major post-translational modifications and protein sorting process efficiently at a pH of 6.2-7. In specific cell types, v-ATPases are also found at the plasma membrane, where they contribute to acidify the extracellular environment.^89^

**Figure 4.**
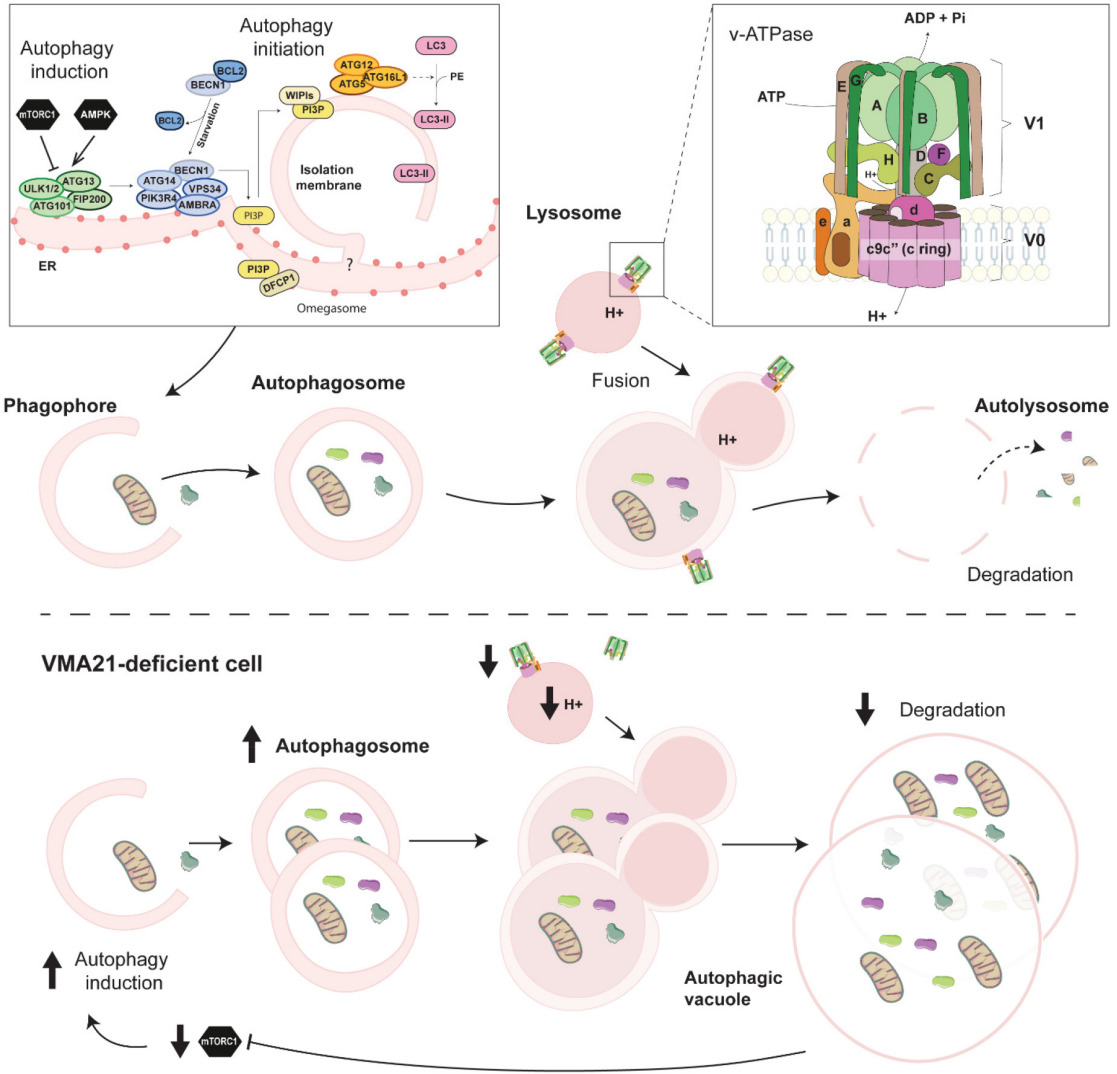
Autophagic flux and autophagy impairment in VMA21-deficient cells. Overview of the autophagy process and of the proteins involved in mammals. Autophagy induction depends on the activation state of mTORC1 and AMPK, which regulate the Ulk1 complex. Autophagic vesicles engulf large cytoplasmic parts, including organelles, which are degraded after fusion with lysosomes and recycled. Defective degradation steps in VMA21-deficient cells lead to the formation of AVSF.

The yeast v-ATPase is a large complex consisting of a cytoplasmic V_1_ domain formed of 16 sub-units (A_3_,B_3_,C,D,E_3_,F,G_3_,H) responsible for ATP hydrolysis, and of a transmembrane V_0_ domain responsible for proton translocation, composed of the subunits a,c_8_,c’,c’’,d,e,f and the recently discovered Voa1p^90,91^ (see Figure 4). The sub-units c, c’ and c” form the c-ring of the V_0_ domain. Mammalian v-ATPases closely resemble yeast v-ATPase organization. However, the sub-units ATP6AP1 and RNAseK replace Voa1p and the sub-unit f in the V_0_ domain. Moreover, the c_8_c’c’’-ring is replaced by the sub-units c_9_c”, with the additional sub-unit ATP6AP2.^92–94^ Finally, CryoEM of the v-ATPase in porcine kidney and rat brain suggest that a small proportion of V_1_ domain may contain an additional C subunit.^93,95^

Interestingly, only the sub-unit V_0_a exists in two isoforms in yeast. In contrast, mammalian v-ATPases have multiple isoforms of each sub-unit, which are expressed in specific tissues, cells or organelles.^88^ For example, the a2 isoform of the subunit V_0_a is included in v-ATPases present in the Golgi and early endosomes, but not in lysosomes. Mutations in *ATP6V0A2*, encoding this specific isoform, cause autosomal recessive cutis laxa type 2 (ARCL2A) characterized by developmental delay, skin hyperelasticity and neurologic abnormalities, and associated with defects in glycosylation and endocytosis.^96,97^ Similarly, mutations in *ATP6V0A3, ATP6V0A4* or *ATP6V1B1,* encoding the osteoclast-specific V_0_a3 subunit, and the isoforms V_0_a4 and V_1_B1 predominantly expressed in kidney and inner ear, cause osteopetrosis, renal tubular acidosis and/or deafness.^98–100^

#### Mechanisms of v-ATPase assembly

Biochemical and genetic analysis in yeast have revealed that Vma21p and two additional chaperone proteins, Vma12p and Vma22p, are required for the assembly of the v-ATPase enzyme, but not included in the fully assembled complex.^90,101–103^ Vma12p and Vma22p are homologous to mammalian TMEM199 and CCDC115. High-resolution cryoEM provided detailed information on how these chaperones cooperate for the assembly of the V_0_ domain in the ER, before it binds V_1_ in the Golgi.^104^ In their recent study, Wang et al. described the structure of V_0_:Vma12p-Vma22p and V_0_:Vma21p complexes.^86^ Vma12p and Vma22p initially bind the subunit V_0_d and the c ring. The subsequent interaction between Vma12p and the subunit V_0_a facilitates the recruitment of V_0_e and V_0_f subunits, thereby completing the assembly of V_0_. Vma22p occupies the binding site of the subunit V_1_D on the sub-unit V_0_d, preventing premature interaction between V_1_ and V_0_ domains. The Vma12/22p complex is released when V_0_ undergoes a conformational change to a lower energy state. At this stage, V_0_ is escorted by Vma21p to the Golgi apparatus.^86^ Vma21p binds to different sites around the c-ring of the immature V_0_ domain: Vma21p preferentially binds V_0_c_2_ and V_0_c_3_, although multiple copies of Vma21p may bind around the c-ring at low density. On top of that, Wang et al. also provided evidence of two yet-unidentified proteins, YAR027W and YAR028W, that interact with the c subunits and with Vma21p.^86^ Although the precise location of these proteins in the c complex remains unclear, this suggests that Vma21p activity may depend on the interactions with other binding partners. Upon reaching the Golgi apparatus, the V_0_ complex binds the V_1_ domain, releasing Vma21p. Due to its retrieval signal, Vma21p cycles back to ER, while the v-ATPase is transported to its final destination.

The role of VMA21 in chaperoning the assembly of V_0_ domain has also been reported in mammals. In human cells, VMA21 was shown to interact with v-ATPase V_0_c’’ subunit.^7^ Esmail et al. (2018) also showed by co-immunoprecipitation that VMA21 binds glycosylated, but not unglycosylated, V_0_a subunit.^105^ The authors conclude that the oligosaccharide moieties may function as a recognition signal for VMA21, which may retain unglycosylated subunits in the ER and prevent their incorporation into V_0_ domain. More recently, we showed that both VMA21 isoforms interact with the V_0_ domain through binding with the c subunit in mouse muscle cells.^84^ Our study also revealed direct or indirect interaction between the two VMA21 isoforms, suggesting synergistic or competing effect towards V_0_ assembly. Finally, in line with a role of VMA21 in chaperoning v-ATPases, VMA21 deficiency in cells from XMEA patients reduced v-ATPase activity, potentially as a result of diminished v-ATPase levels. V_1_E subunit accumulated in the cytosolic fraction, suggesting decreased formation of the V_0_/V_1_ complex.^7^

### *VMA21* circRNA

Apart from VMA21 proteins, recent evidences emerged regarding the existence of circRNAs encoded by *VMA21*. CircRNAs are a specific type of non-coding RNA, which are single-stranded and covalently closed biomolecules without 5′ or 3′ ends. They are evolutionarily conserved across species, from viruses to mammals, and can arise from exons (exonic circRNA) or introns (intronic circRNA).^106^ CircRNAs can act as protein decoys and scaffold for protein interaction. In addition, circRNAs harbour multiple binding sites for miRNAs, thereby serving as inhibitors of miRNAs.^107^ circRNAs unique expression signature has been involved in cancer,^108^ autoimmune^109^ and cardiovascular diseases.^110^

In recent years, an increasing number of studies pointed to the involvement of *circVMA21* in inflammatory conditions, through inhibition of specific miRNAs. In a rat model of intervertebral disc degeneration, *circVMA21* targeted miR-200c/XIAP (X-Linked Inhibitor Of Apoptosis) pathway and alleviated inflammation and apoptosis.^111^ Similarly, *circVMA21* reduced inflammation in different models of sepsis and kidney injury, by sponging miRNAs, such as miR-497-5p and miR-7-5p.^112,113,114,115,116^ Recent findings also revealed chondroprotective effects of *circVMA21* via acting on miR-495-3p/FBWX7 (F-Box And WD Repeat Domain Containing 7)^117^ and miR-103,^118^ providing valuable directions for the treatment of osteoarthritis. Overall, decreased expression of *circVMA21* associated with increased inflammation in all pathologies, while its overexpression relieved inflammatory injury, pointing to *circVMA21* as a promising therapeutic target. It is noteworthy that current evidence also suggests that circRNAs can code uncharacterized proteins with high sequence homology compared to mRNA-encoded isoforms. This makes them attractive for a competitive binding to partners of cognate proteins, in what is known as the “protein bait hypothesis”.^119^ Whether *circVMA21* perturbs the activity of VMA21 proteins and/or participates in the progression of XMEA, *VMA21*-CDG or lymphoma still needs to be studied.

## VMA21-associated pathomechanisms: from cellular functions to pathogenesis

### Autophagy impairment

#### Regulation of the autophagic flux

Autophagy is an intracellular degradation process in which cytoplasmic materials and organelles are degraded in lysosomes. Among other types of autophagy, macroautophagy (hereafter simply referred to as autophagy) ensures basal elimination and recycling of defective organelles or misfolded proteins. Its induction increases in stress conditions conferred by oxygen, nutrient or growth factor deprivation.^120,121^ Autophagy involves the formation of a phagophore that engulfs targets and elongates to form a double-membrane vesicle, called autophagosome.^122^ The fusion of autophagosome with lysosomes leads to autolysosome, in which lysosomal enzymes degrade the autophagic content (Figure 4). The autophagic flux is hence determined both by the state of autophagy induction and the efficiency of the degradation steps.^120,121^

Proteins encoded by autophagy-related genes (Atg), as well as other key factors, such as the cargo receptor p62, tightly orchestrate autophagy. The extent of autophagosome formation is mainly determined by mTORC1 and 5′ AMP-activated protein kinase (AMPK), which inhibits and increases autophagy induction, respectively (Figure 4). In growth conditions, activation of mTORC1 leads to phosphorylation and inhibition of the serine/threonine kinase Ulk1, involved in autophagy initiation.^123,124^ Contrarily, under starvation or other stress conditions, mTORC1 inhibition releases Ulk1 activity and triggers autophagy induction. In these conditions, AMPK activation contributes to the inhibition of Raptor, one of the components of mTORC1, and activates Ulk1 by phosphorylating serines 317 and 777.^125–127^ Although precise evaluation of the autophagic flux represents a limitation in humans, there is increasing evidence that its perturbation contributes to a large spectrum of diseases.^121^ In skeletal muscle, insufficient autophagic flux, conferred by blockade of the induction or degradation steps, as well as excessive autophagic flux, trigger loss of muscle homeostasis, muscle atrophy, and potentially muscle degeneration.^128–130^ Autophagy impairment emerged as one of the main contributor of many myopathies,^10^ and several evidence suggest that it is central in the pathomechanisms leading to VMA21-associated diseases.

#### Evidence of autophagic dysregulation caused by VMA21 deficiency

Acidification of the lysosomal lumen is decisive for the activity of lysosomal enzymes, and thereby for the degradation steps of autophagy. Nakamura and al. first demonstrated the importance of v-ATPase-mediated acidification in autophagic degradation in yeast.^131^ They showed that *vma* mutants (*i.e.,* deficient for v-ATPase) accumulate autophagic bodies in the vacuole resulting from defective degradation. In particular, yeast lacking Vma21p do not contain acidified vacuoles and exhibit similar phenotypes as other *vma* mutants.^83^ In line with this, inhibition of lysosomal function with bafilomycin A, a v-ATPase inhibitor, prevented luminal acidification and lysosomal enzyme activity, with a robust block of autophagic flux in *Drosophila.*^132^

The first evidence of autophagy impairment in XMEA is the accumulation of autophagic vacuoles in muscle biopsies from patients. These vacuoles stain positive for the lysosomal marker LAMP2, and for the autophagic markers p62 and LC3, which confirm their autophagic nature.^32,33,40,46^ Electron microscopy further confirmed the accumulation of autophagosomes and vacuoles containing cell debris in skeletal muscle, as well as in fibroblasts, from XMEA patients.^19,21,23–26,38–40,42,43^ In lymphoblasts from XMEA patients, Ramachandran et al. (2013) evaluated the remaining v-ATPase activity at 12–22% of control cells, which was accompanied by higher lysosomal pH (pH 5.2 vs. 4.7).^7^ In parallel, the elevation of LC3II and p62 levels in patient's fibroblasts and lymphoblasts was consistent with a blockade in the degradation steps of autophagy and reduced autophagic flux.^7^ Interestingly, patient cells also displayed major increase in Beclin-1 and Vps34 levels, as well as reduced phosphorylation of mTORC1 targets. Consistently, they uncovered a reduction in intracellular free amino acids that may arise from the partial block of autophagy. The authors hence suggested that the primary autophagy blockade, by reducing amino acid levels, triggers mTORC1 inhibition, and thereby increases autophagy induction. This feedback may exacerbate the accumulation of autophagic vesicles and vacuoles in cells from XMEA patients (Figure 4).

Cannata Serio et al. (2020) also found v-ATPase dysfunction in fibroblasts from *VMA21*-CDG patients, as well as reduced lysosomal acidification.^8^ Increased p62 and LC3 levels suggested autophagic blockade in fibroblasts from *VMA21*-CDG patients, similar to that observed in XMEA patients. Notably, autophagic defects were associated with an accumulation of lysosome-associated structures and an alteration of lysosomal morphology in *VMA21*-CDG fibroblasts. Electron microscopy confirmed that autolysosomes accumulate in Kupffer cells and hepatocytes in a liver biopsy of a *VMA21*-CDG patient.^8^

Interestingly, *VMA21* variants associated with FL also led to autophagy blockade,^9^ similar to XMEA and *VMA21*-CDG. Electron microscopy showed an accumulation of autolysosomes in LF cells mutated in *VMA21*. As shown in XMEA cells, there was also a compensatory increase in autophagy induction in these cells. The role of autophagy in cancer cells is controversial. Many studies suggest that autophagy may prevent malignant transformation, whereas other studies have shown that autophagy is involved in cancer development, metastasis and cancer-drug resistance.^133,134^ Wang et al. suggested that increased autophagic induction in *VMA21*-mutated cells may be a key factor determining survival for tumour cells, which may be pharmacologically targeted in therapeutic strategies.^75^ Accordingly, targeting HEK293T cells harbouring *VMA21* mutations with a drug inhibiting autophagy induction effectively killed these cells, pointing to novel therapeutic strategies for FL associated with aberrant v-ATPase activity. However, these strategies should be adapted depending on the tissue and cancer type. Indeed, as mentioned above, VMA21 may function both as a tumour suppressor and tumour promotor. Whether the role of VMA21 in modulating tumorigenesis only relies on its effect on the autophagic flux remains unclear.

#### Therapeutic strategies

Since reduced levels of *VMA21* in XMEA and CDG cells blocks autophagic degradation and in turn increases autophagy induction, it becomes challenging to propose a straightforward approach based on autophagy modulation as a therapeutic strategy. Ideally, this strategy would limit autophagy induction and restore lysosomal pH with acidifying agents, in order to prevent accumulation of autophagic vesicles. Lysosomal re-acidification has been proposed as a potent approach for LSDs.^13^ Biodegradable acidic nanoparticles restored lysosomal acidity and autophagy in mouse models of non-alcoholic fatty liver disease (NAFLD).^135^ These nanoparticles remain intact and inactive at plasma pH, while they get degraded around pH 6, which may correspond to the luminal pH of lysosomes in the case of impaired acidification. Acidic nanoparticles also improved lysosomal function and the phenotype of mouse models of neurodegenerative disorders, such as Parkinson's or Alzheimer's diseases (AD).^136,137^ Interestingly, presenilin1 (PS1) involved in AD interacts with the subunit V_0_a1 of v-ATPase, and thereby regulates its delivery to lysosomes.^138^ PS1 deficiency disrupted this interaction, reduced accumulation of functional v-ATPase in lysosomes, and impaired lysosomal function in the brain of AD mouse models. However, these defects were not reproduced in cells or mouse brain deficient for PS1/2.^139^ Modulating v-ATPase activity with specific compounds, such as dendrobium alkaloids, enhanced lysosomal acidification and improved learning function in β-amyloid precursor protein (APP)/PS1 double transgenic mice.^140^ In line with this, targeting V_1_A subunit with the small molecule EN6 effectively cleared toxic Tar-DNA binding protein 43 (TDP-43) aggregates in a cell line model of amyotrophic lateral sclerosis (ALS) pathogenesis. EN6-mediated clearance of protein aggregates involved mTORC1 inhibition, which subsequently enhanced the nuclear translocation of transcription factor EB (TFEB), increased the expression of genes encoding v-ATPase subunits, and ultimately improved lysosomal acidification.^141^ Similarly, overexpressing TFEB in muscle cells or in mouse models for Pompe disease reduced lysosomal size and glycogen accumulation, and enhanced cellular clearance by promoting lysosomal exocytosis.^142^ Of note, inhibition of autophagy induction by targeting Atg5 / Atg7 increased the benefits of the enzyme replacement strategy in mouse models of Pompe disease.^143^ These recent pharmacological developments open promising perspectives for the restoration of lysosomal acidification in skeletal muscle and/or liver in patients suffering from XMEA and CDG. However, it remains unknown whether the normalization of lysosomal function and autophagy will be sufficient to reverse muscle/liver phenotypes, as XMEA/CDG pathogenesis may involve other VMA21-dependent processes.

### Glycosylation defects

N- and O-glycosylations are prevalent post-translation modifications occurring within the Golgi apparatus.^144,145^ N-glycosylation generally corresponds to the attachment of a glycan to a nitrogen of an asparagine residue within the consensus sequence Asn-X-Ser/Thr, with X representing any amino acids except proline. In O-glycosylation, the glycan is attached to the hydroxyl oxygen of a serine or a threonine residue. Glycosylation has been identified on more than 3000 proteins.^146^ As mentioned above, CDGs are typically severe multisystemic disorders, involving different glycosylation defects.

CDG patients with *VMA21* mutations showed N- and O-glycosylation abnormalities with marked accumulation of truncated glycans lacking sialic acid and galactose.^8^ Interestingly, mutations in genes encoding the other chaperones of the v-ATPase, CCDC115 and TMEM199, or the associated proteins ATP6AP1/2, led to similar combined N- and O-glycosylation defects and to hepatopathy.^66,67,69,70^ Mutations in the gene encoding v-ATPase V_0_a2 subunit have also been linked to impaired glycosylation, which may be key in the pathogenesis of cutis laxa type II syndrome.^96^ The glycosylation defects reported in these disorders are typically occurring in the *trans* Golgi. Increase in trans Golgi pH, caused by v-ATPase deficiency, likely alters the activity of enzymes involved in the last glycosylation steps. In line with this, neutralization of Golgi pH with bafilomycin A impaired glycosylation and resulted in the delocalization of glycosylation enzymes.^147^ Notably, mutations in *ATP6V0A2* altered the retrograde translocation and fusion of membranes from Golgi to ER in fibroblasts.^96^ This defective trafficking may also contribute to glycosylation perturbations in patients. However, the morphology of Golgi and ER organelles was preserved upon bafilomycin A treatment, suggesting that v-ATPase activity is not essential for Golgi/ER network regulation.^147^ Similarly, cells with mutations in *ATP6AP1/2*, *CCDC115* or *ATP6V0A2* showed normal Golgi structure.^68–70,96^ In contrast, VMA21 and TMEM199 deficiency led to dilated Golgi in hepatocytes.^8,67^ Hence, genetic deficiency in v-ATPase assembly factors is associated with glycosylation defects, but whether these defects arise from impaired Golgi acidification has not been thoroughly investigated. It is noteworthy that protein glycosylation was measured in three different XMEA patients and found to be normal.^8,148^

### Lipid metabolism dysregulation

Several evidences suggest that VMA21-associated diseases involve dysregulation of lipid metabolism. Liver plays central functions in the capture, storage, transformation and release of lipids. Free fatty acids (FFAs) captured by hepatocytes are used in β-oxidation, or alternatively converted into triglycerides (TG) and stored in lipid droplets (LD) or released in the circulation as very low-density lipoproteins (VLDL). Cholesterol from blood, included in low-density lipoprotein (LDL), enters in hepatocytes, where it can be stored in LDs as cholesteryl esters. A tight regulation of cholesterol storage is required to maintain homeostatic levels of free, potentially toxic, cholesterol in cells.^148^ In this context, lysosomes play a major role in regulating the intracellular trafficking and hydrolysis of cholesterol.^149,150^ Endocytosed cholesterol-LDL are delivered to lysosomes, where lysosomal acid lipases hydrolyse esterified cholesterol into free cholesterol. Notably, the dissociation of cholesterol-LDL from LDL receptors after internalization requires proper acidification of the endosomes. From the lysosomal lumen, cholesterol is then conveyed to the lysosomal membrane through interaction with different proteins, including Niemann-Pick C (NPC) 1/2 proteins, as well as LAMP2 and lysosomal integral membrane protein (LIMP2).^151–153^ These interactions ensure the transport of cholesterol to other organelles, such as the ER, as well as to the plasma membrane. In the NPC lysosomal storage disease, NPC1/2 deficiency leads to the accumulation of cholesterol and other lipids in lysosomes and endosomes of most cells, causing neurodegeneration and dysfunction of lung and liver.^154^

Lysosomes are also key in lipid hydrolysis ensured by autophagy, *i.e., “*lipophagy”.^155^ Activity of lysosomal acid lipases involved in lipophagy requires low pH.^156^ In hepatocytes, lipophagy contributes to the degradation of intracellular LDs and to the release of FFA. In line with this, patients with *TMEM199* and *CCDC115* mutations present hypercholesterolemia and fatty liver disease.^157^ Impaired lipophagy also seems involved in *VMA21*-CDG: hepatocytes from patients show intracellular accumulation of LDs, which can be found in autolysosomes or lysosome-like structures.^8^ LD accumulation was also reported in XMEA fibroblasts, although LD-containing autolysosomes have never been described.^8^ Fibroblasts from *VMA21*-CDG patients and, to a lesser extent, from XMEA patients, also exhibited a build-up of cholesterol.^8^ Bafilomycin A treatment exacerbated LD and cholesterol accumulation in control and XMEA fibroblasts. In contrast, bafilomycin A did not further increase LD and cholesterol accumulation in *VMA21*-CDG hepatocytes.^8^ This suggested different pathomechanisms depending on the mutation in *VMA21* and/or the cell type. Of note, cholesterol retention in lysosomes reduced its amount in the ER, secondarily promoting the lipogenic pathway: levels of sterol response element-binding protein-1 (SREBP1), a sensor of cholesterol in ER, increased in *VMA21*-CDG but not in XMEA fibroblasts. Upregulation of SREBP1-mediated lipogenesis may hence contribute to the elevated levels of plasma cholesterol in *VMA21*-CDG patients.^8^

### ER stress

ER stress arises from an accumulation of unfolded or misfolded proteins in the ER, which usually results from an imbalance between protein synthesis, maturation and intracellular trafficking.^158^ The pathogenic role of ER stress has been established in several disorders, such as neurodegenerative diseases, diabetes and liver cirrhosis.^159–161^ ER stress, in particular in muscle fibres, can trigger autophagy induction, which may then contribute to the elimination of unfolded proteins.^162–164^ However, ER stress also reduced lysosomal function in muscle fibres,^164^ suggesting that it may rather limit autophagic flux. Of note, reduced levels of VMA21 were detected upon ER stress induction in muscle fibres.^164^

In CDG fibroblasts, VMA21 deficiency resulted in activation of the unfolded protein response (UPR) associated with protein kinase R-like ER kinase (PERK), suggesting ongoing ER stress.^8^ Deficiency for the chaperone ATP6AP2 in *Drosophila* also led to ER stress, which may arise from the defective assembly of the v-ATPase V_0_ domain and its potential degradation within the ER.^165,166^ Whether VMA21 chaperones the assembly and/or folding of other proteins or complexes in the ER remains unknown. In this context, VMA21 deficiency may induce ER stress by dysregulating the maturation of several proteins, including v-ATPase V_0_ domain. Although ER stress and UPR activation were not detected in fibroblasts from one XMEA patient analyzed by Cannata et al.,^8^ they remain to be investigated in muscle cells from XMEA patients.

## Conclusion

Interest to VMA21 has grown these last years with the expansion of the clinical spectrum associated with *VMA21* mutations. The reported role of VMA21 in the assembly of the v-ATPase pump is consistent with pathogenic involvement of autophagy, glycosylation, lipid metabolism, and/or ER stress in XMEA and *VMA21*-CDG. The comparison between XMEA and *VMA21*-CDG uncovered differences in the clinical features and pathomechanisms of the two diseases. While ER stress and glycosylation defects were not observed in XMEA patients, impaired autophagy and increased cholesterol were detected in both *VMA21*-CDG and XMEA cells. Residual levels of VMA21, and whether one or the two VMA21 isoforms are affected by the mutation, may determine the processes impaired and/or the tissue clinically targeted. Hence, further investigations are required to clarify the respective contribution of VMA21 isoforms in the pathogenesis of XMEA and *VMA21*-CDG, and the pathomechanisms underlying the clinical differences between the two diseases. In line with this, systematic evaluation of the expression of VMA21-120 (in addition with VMA21-101) in patients with CDG or XMEA, as well as the identification of other targets/partners of each isoform, are crucial. The role of circ*VMA21* in XMEA and *VMA21*-CDG should also be considered in this puzzle. Future research development on these mechanistic aspects will ultimately help designing common or specific therapeutic interventions for *VMA21*-related diseases.

## Abbreviations

AD: Alzheimer Disease
ALS: Amyotrophic Lateral Sclerosis
ALT: Alanine aminotransferase
AMPK: AMP-activated protein kinase
AST: Aspartate aminotransferase
AVM: Autophagic Vacuolar Myopathies
AVSF: Autophagic Vacuoles with Sarcolemmal Features
CDG: Congenital Disorder of Glycosylation
circRNA: Circular RNA
CK: Creatine kinase
CRC: Colorectal cancer
ER: Endoplasmic reticulum
ERGIC: ER-Golgi intermediate compartment
FFA: Free fatty acid
FL: Follicular Lymphoma
GAA: Acid alpha-glucosidase
ICGC: International Cancer Genome Consortium database
LAMP2: Lysosomal Associated Membrane Protein 2
LD: Lipid droplet
LDL: Low-density lipoproteins
LIMP2: Lysosomal Integral Membrane Protein 2
lncRNA: long non-coding RNA
LPS: Lipopolysaccharide
LSD: Lysosomal Storage Disorder
LUAD: Lung adenocarcinoma,
mTORC1: mammalian Target of Rapamycin Complex 1
NAFLD: Non-Alcoholic Fatty Liver Disease
NPC: Niemann-Pick C
PS1: Presenilin 1
TFEB: Transcription Factor EB
TG: Triglyceride
THPA: The Human Protein Atlas
TSS: Transcription start site
UPR: Unfolded protein response
v-ATPase: Vacuolar ATPase
VLDL: Very low-density lipoprotein
XMEA: X-linked Myopathy with Excessive Autophagy
